# Working Women in Large Towns. IV

**Published:** 1890-06-21

**Authors:** 


### Working Women in Large Towns.—IV.

### Class II.—Factory and Work-room Hands (*continued*).

### The City Work-girl.

This is another genus of the order Factory-girls. The City work-girl would strongly object to be classed with the girls whom we have considered in our last article, although in practice it often happens that the two get to some extent mixed; and their mode of life is really much the same, except that the City work-girl considers herself, and indeed is, a grade or two higher in the social scale. While the father of the factory-girl is generally a labourer, the father of the City work-girl will be an artisan—even a country artisan, for it is a melancholy fact that country girls will persist in thinking that they have only to come to London to make their fortune, and hurry up to swell the numbers of those who are already hard pressed by competition; sometimes getting work, indeed, but too often taking the downward course to which despair and hunger seem to drive them. When they do find work, they join the ranks of fancy trimming-makers, box-makers, bookbinders, button-makers, muff-liners, spectacle-makers, straw-workers, or bag-makers—to give a few specimens of the numerous trades in which City work-girls are employed. They earn considerably more than the factory girl proper; but then their standard of living is higher, and their slack times reduce their wages to from eight to fourteen shillings a week. They consider themselves "young ladies," and feel it incumbent upon themselves to make a smart appearance; indeed, their employers would probably have something to say to the shabby garments which would better match the slenderness of their purses; and it is to be feared that many of them look to a trade which can only be hinted at here, for the supply of their luxuries, amusements, and sometimes even of the necessaries of life. "Gentlemen are the only people who speak to me civil," was said to a commissioner of the *British Weekly* by a poor girl who lived by herself in a wretched garret, and had been out of work *nearly six months.* It is comparatively easy for London girls, whose friends will help them during slack times, to keep straight; but what is the country girl to do when she finds herself out of work, out of money, and with no respectable means of earning any more for weeks, perhaps even for months to come? The religious world must surely have been startled to hear from the author of *Toilers in London* that while bookbinding generally is poorly paid, the book-sewers employed by religious publishers are among the very worst paid of our City work-girls; truly this throws a terrible side-light upon the vaunted "cheapness" of our Bibles and tracts!

The employers of these girls are, one and all, forbidden under the Factory Acts to work their hands for more than twelve hours, allowing a certain time for meals out of that; though it may be mentioned, by the way, that the law is often evaded—and that with the connivance of the girls, who are delighted to earn "overtime" pay. But there is no such protection for the next group of women whom we have to consider, though we have placed them in this class because the other conditions of their work appear to be much the same—indeed, it is difficult to suppose that they were left out of the Factory Acts by anything but a blunder.

### Laundresses.

On the whole, laundresses are fairly well paid. Those who "dress" collars and cuffs for the large warehouses can earn four or five shillings a-day; but then the work is hard and must be done well, and laundry women hardly ever get more than four or five days' work in the week. Washers of soiled linen earn two-and-sixpence a day in the metropolitan districts, while ironers can make from three-and-six to five shillings; but they also never work all the week, and it must be remembered that they have to stand all day at their work in an atmosphere heated with steam and gas, and terribly oppressive. It is no uncommon thing for a young laundress to faint from heat and fatigue; and when at last work is over for the day, the change from the over-heated room to the outside air, coming suddenly to poor creatures wearied out and damp with perspiration, chills them through and through, and often sows in them the seeds of consumption, to be developed later on. As for long hours, it is said that in the steam-laundries, where many children as well as girls are employed, work is often kept up from seven in the morning till ten at night.

There is now a thriving Laundresses' Union at Fulham, which has several branches, numbering over four hundred members. It is to be hoped that it may do something towards shortening the hours of labour of these women, who, like so many of the poor, take their troubles too much as a matter of course, and hardly seem to have realised the cruelly long hours which they were working, until it was pointed out to them by outside friends.

### Boot and Shoe Makers.

Large numbers of women and girls are engaged in this trade, not in London only, but in Leeds, Leicester, and various other provincial towns. To men-workers is always entrusted the task of making soles, heels, &c., and cutting out the "uppers" of boots and shoes; but these uppers, when cut out, are given to women-workers to be fitted, machined, and button-holed; and the work of "trimming" shoes, *i.e.*, putting on bows and buckles, is also entrusted to them. In the making of "sew-round goods"—in other words, fancy shoes and slippers, women are also largely employed, but they as a rule work at home. Those whom we have now to consider either work in large factories or in small workshops. The former may be said to be in fairly comfortable circumstances, for the rooms in which they work are large and airy, and the best hands can make from fifteen to twenty shillings per week; moreover, their work necessitates considerable training, and there is, therefore, less competition in it than in many trades. But the case of many girls working in small work-rooms is far different. These work-rooms are in the most crowded parts of Bethnal Green and Spitalfields; they are always small, often underground, and, when we add that they are not unfrequently used as sleeping-rooms, it can be no matter of surprise that many of them are not only dark, but filthy. The wages of these workers are also very low—even leaving out of consideration the fact that the slack season lasts from November till March. Mr. D. Schloss mentions the case of a female "sewer," working with several men in a small workshop, whose weekly wage may be estimated at about sixteen shillings. At the same time, he adds that the head of this workshop had no work for his hands from Christmas till Easter; and bearing in mind that this slack season was a rather exceptionally short one, it is terrible to reflect on the straits to which others, who earn less, must be reduced. That numbers of them do earn less is indisputable. Here is the statement, not of a sensational, screamy newspaper, but of a steady-going factory inspector, Mr. Lakeman: "An ordinary machinist can earn nine to ten shillings a-week, but one who works on best goods goes up to twelve, fourteen, and fifteen shillings: a good fitter makes twelve shillings a-week, whilst hand-workers must be content with seven or eight shillings." Comments are needless.

